# From Fear to Hopelessness: The Buffering Effect of Patient-Centered Communication in a Sample of Oncological Patients during COVID-19

**DOI:** 10.3390/bs11060087

**Published:** 2021-06-14

**Authors:** Alessandro Alberto Rossi, Maria Marconi, Federica Taccini, Claudio Verusio, Stefania Mannarini

**Affiliations:** 1 Department of Philosophy, Sociology, Education, and Applied Psychology, Section of Applied Psychology, University of Padova, 35131 Padova, Italy; federica.taccini@phd.unipd.it (F.T.); stefania.mannarini@unipd.it (S.M.); 2 Interdepartmental Center for Family Research, University of Padova, 35131 Padova, Italy; 3 Department of Medical Oncology, Presidio Ospedaliero di Saronno, ASST Valle Olona, 21047 Saronno, Italy; maria.marconi@asst-valleolona.it (M.M.); claudio.verusio@asst-valleolona.it (C.V.)

**Keywords:** psycho-oncology, oncology, fear, hopelessness, communication, patient-centered communication, quality of life, COVID-19

## Abstract

Background: COVID-19 represents a threat both for the physical and psychological health of oncological patients experiencing heightened distress levels to which the fear of the virus is also added. Moreover, fear of COVID-19 could lead oncological patients to experience feelings of hopelessness related to their medical care. Patient-centered communication may act as a buffer against the aforementioned variables. This study aimed to test the role of doctor–patient communication in the relationship between fear of COVID-19 and hopelessness. Methods: During the COVID-19 pandemic, a sample of 90 oncological outpatients was recruited (40 males (44.4%) and 50 females (55.6%), mean age = 66.08 (*SD* = 12.12)). A structured interview was developed and used during the pandemic to measure the patients’ perceived (A) fear of COVID-19, and (B) feelings of hopelessness, and (C) physicians’ use of empathetic and (D) clear language during the consultation. A multiple mediation model was tested, and the effects between males and females were also compared. Results: Empathetic and clear doctor–patient communication buffered the adverse effect of the fear of COVID-19 on hopelessness through a full-mediation model. The effects did not differ between males and females in the overall model but its indirect effects. Discussions: Patient-centered communication using empathy and clear language can buffer the adverse effect of the fear of COVID-19 and protect oncological patients from hopelessness during the pandemic. These findings might help to improve clinical oncological practice.

## 1. Introduction

In March 2020, due to the global spread of the coronavirus disease (COVID-19), the World Health Organization declared the situation as a pandemic [1,2,3].

The COVID-19 virus can rapidly spread and it causes a potentially mortal acute respiratory syndrome [1,2,4,5,6,7].

Although effective preventive policies were adopted by countries worldwide (i.e., social isolation, social distancing, school closures, reduction or suspension of economic activities, and curfew [8,9,10]), the COVID-19 epidemic continued to demonstrate a growing pattern of community transmission. Italy was one of the most affected countries during the outbreak, initially accounting for over 223.000 individuals infected by COVID-19 and more than 31.000 deaths [11]. In May 2021, 4.111.110 people resulted having contracted the virus, and 122.833 of them died as a consequence [12].

Due to comorbidities with other medical conditions, some people were found to be at increased risk of morbidity and mortality for COVID-19 [13,14], including elderly [15,16,17,18,19], individuals with obesity [20,21,22,23], or cardiac diseases [24,25,26,27], and persons with respiratory problems [28,29], Particularly, oncological patients have been reported to be at increased risk of infection, and a more severe disease course [30,31,32,33], with a large proportion of people requiring high levels of intensive care, having a more rapidly evolving disease, and having an increased risk of death [34,35,36].

The awareness of the increased vulnerability to severe COVID-19 syndrome, together with the lived experience of COVID-19 and its related consequences, have intensified both psychological difficulties and negative feelings already existing in oncological patients [34,35,37,38].

In fact, as a consequence of generic advice given to people who are clinically susceptible to COVID-19, patients with cancer (of any age, gender, tumor subtype, and stage) had to deal with several changes in the management of their condition during the past few months [39,40,41] including shortening of radiotherapy [42,43], delays in therapies, modifications of therapeutic regimens [44,45] and restrictions of visits from their loved ones [37,45,46,47] with consequent reductions in socio-emotional support [48,49]. All these circumstances contributed to intensifying the burden of negative emotions experienced by patients with cancer [50,51], thus representing a hazardous trigger for fearful response characterized by worries for their health [6,43,50,51].

Consequently, regarding the contextual factors concerning the delay of medical treatments, COVID-19 may represent a hazardous trigger for oncological patients. Research further shows that fear of COVID-19 might be associated with feelings of hopelessness [9]. Indeed, 17% of oncological patients reported experiencing fear related to the COVID-19 virus [45] as well as worries about a possible contagion [34,35], and the impact it might have on cancer treatment (i.e., delays in therapies, etc.) [52]. This resulted in an increased feeling of hopelessness about the future in about 13% of cancer survivors during the pandemic [37], which represents an important risk factor for the development of depressive symptoms [9,53]. Beyond psychological health, negative feelings may affect patients’ adherence to cancer therapies, and therefore their physical health [35,37,45,54,55].

Still, several studies suggest that a good doctor–patient relationship might act as a protecting (buffering) factor against cancer-related negative feelings, thus preventing the patients from the onset of further psychological issues and other medical issues related to non-adherence to treatment recommendations [56,57,58,59]. In particular, patient-centered communication (PCC) might buffer the negative association between fear of COVID-19 and hopelessness by (1) fostering the patient–clinician relationship; (2) exchanging information; (3) responding to emotions; (4) managing uncertainty; (5) supporting decision-making processes and (6) enabling patient self-management [57,60,61,62] thus promoting better patients’ psychosocial adjustments [57,58,59,60,63].

Particularly, two important aspects seem to play a central role in PCC: empathy and clear language [64,65,66,67,68].

Indeed, physicians’ ability to communicate with patients by expressing validation, empathy, and support [57] seems to contribute to a better doctor–patient relationship and higher satisfaction with the consultation [69]. Furthermore, patients’ perceived empathy positively influences their psychological well-being: when doctors empathically acknowledge patients’ feelings and encourage them to pursue the goal of the treatment, patients show decreased anxiety symptoms and increased trust in the doctors’ recommendations [70]. In addition, Epstein (2007) highlighted that, according to PCC, doctors should ask patients what information they need, provide them with clear answers, then check the patients’ understanding of the shared information. Indeed, sharing clear information is one of the communication strategies that mostly contribute to reducing patients’ anxiety [57]. Additionally, the clarity of information helps one to face possible feelings of uncertainty, enhancing patients’ psychological well-being [62].

Clinician–patient communication presenting both empathy and clarity of language seems to calm oncological patients’ fears and to reduce both their anxiety symptoms and their feeling of hopelessness [58,62,71,72,73,74].

Still, the research focused on the impact of doctor–patient communication on oncological patients’ negative feelings at the time of COVID-19 is scant [34,72,75], and no study has previously investigated the relationships between fear of COVID-19 and feelings of hopelessness in this patient population.

In this scenario, the present research aimed to examine “if” and “how” two of the main characteristics of doctor–patient communication (i.e., empathy and clarity) might act as mediators (with a buffering effect) in the relation between fear of COVID-19 and hopelessness in a sample of oncological outpatients during the outbreak. Specifically, it was hypothesized that an empathetic and clear doctor–patient communication might buffer the relation between fear related to the COVID-19 pandemic and the consequent feeling of hopelessness in patients with cancer.

## 2. Method

An observational research design was used to investigate the psychological experiences that the oncological outpatients made of the COVID-19 pandemic at the beginning of the lockdown (from the third week of March 2020 to the second week of April 2020).

### 2.1. Sample Size Determination

The sample size was determined a priori by considering the statistical analyses used in this study (see designated section). In particular, the “*n:q* criterion” was used where *n* is the number of participants in the study and *q* is the number of model parameters to be estimated [76,77,78,79]. Thus, a minimum of 5 participants per parameter was guaranteed (i.e., 5 participants * 12 parameters) leading to a minimum sample size of 60 participants.

### 2.2. Participants

An initial sample of 100 oncological outpatients was consecutively recruited at admission at the Department of Medical Oncology, Presidio Ospedaliero di Saronno, ASST Valle Olona, in Saronno (VA), Italy.

Inclusion criteria for participating in the study were: (A) being over 18 years old; (B) being a native Italian speaker; (C) having received a diagnosis of cancer within the last 6 months; (D) having had a confrontation with the physician about cancer management during the COVID-19 pandemic; (E) providing signed informed consent. Participants were excluded from the study if they were: (F) unable to attend the clinical interview due to cognitive or speech impairments, and/or to upcoming medical commitments; (G) presenting critical/severe anxiety and/or depressive feelings; or (H) showing unusual physical distress/suffering. Moreover, each participant was an oncological outpatient, he/she should never be hospitalized for problems related to cancer and he/she should follow an intravenous therapy for cancer.

According to the abovementioned criteria, 10 outpatients were excluded from the study.

The final sample comprised 90 participants (40 males (44.4%) and 50 females (55.6%), aged from 30 to 89 years (*mean* = 66.08, *SD* = 12.12, *median* = 67)). Considering the type of cancer, 32 patients had lung cancer (35.6%), 24 patients had breast cancer (26.7% females), 18 patients had gastrointestinal cancer (20%), 11 patients had urogenital cancer (12.2%), and 5 patients had oncohematological cancer (5.6%). Considering education level, 51 patients had a middle school diploma (56.7%), 32 patients had a high school diploma (35.6), and 7 patients had a bachelor/master’s degree (7.8%). Considering civil status, 71 patients were either in a relationship or married (78.9%), 10 patients were either separated or divorced (11.1%), 7 patients were widowed (7.8%), and 2 patients were single (2.2%). Considering working status, 47 patients were retired (52.2%), 24 patients were dependent workers (26.7%), 13 patients were entrepreneurs (14.4%) and 6 patients declared “other” (6.6%).

### 2.3. Measures: (Development of) the Structured Interview

In line with previous research [8,44,80], due to the impossibility of using routinely paper-and-pencil assessment questionnaires, an ad hoc structured interview was created for this study and administered to oncological outpatients. The use of a structured interview was deemed appropriate to allow the investigation of the patients’ thoughts, emotions, and psychological issues using standardized methodological procedures [8].

In line with previous studies [80], the pool of items of the structured interview was developed using a two-step procedure [46,81,82,83,84].

First, two expert psychologists in the field (authors A.A.R. and M.M.) independently conceived and listed 20 ad hoc items investigating 4 specific domains (4 items per domain) related to COVID-19 and PCC.

Second, the two lists of items were merged and screened: item wordings were adjusted to the target population, and redundant items were removed. Then, a final list of 12 items (3 items per domain) was created and approved by the two expert psychologists. The final set of items were rated on a dichotomous scale: 0 (= false) and 1 (= true). No reverse items were retained.

In more detail, the first and the second set of items aimed to investigate negative psychological feelings related to the medical condition of the patients (i.e., oncological disease) within the pandemic framework; namely, (I) the fear of COVID-19 and (II) hopelessness. The third and the fourth set of items aimed to investigate the patient’s perception concerning the communication he/she had with the physician regarding his/her oncological treatment; namely, (III) (perceived) empathic communication and (IV) (perceived) clarity of information received.

Fear of COVID-19

The first set of three items aimed to evaluate the presence (or absence) of fear towards COVID-19 in comorbidity with the oncological problem (i.e., “*Considering your oncological disease, are you afraid of COVID-19?*”). High values indicated high fear of COVID-19. For the present study, the expected a posteriori (EAP) reliability coefficient was equal to 0.715 and the KR-20 was equal to 0.878.

Hopelessness

The second set of three items aimed to evaluate the presence (or absence) of hopelessness towards COVID-19 in comorbidity with the oncological problem (i.e., “*Considering your oncological disease, did the future seem hopeless to you?*”). High values indicated high hopelessness. For the present study, the EAP reliability coefficient was equal to 0.622 and the KR-20 was equal to 0.878.

(Perceived) Empathic communication

The third set of three items aimed to assess the patient’s perception of empathy shown by the physician in communicating care management during the pandemic (i.e., “*Was the doctor empathetic in communicating the management of cancer care during this period?*”). Moreover, considering a possible difficulty in fully understanding the term “empathic” for some patients, a brief explanation of the term was provided by the psycho-oncologist during the interview. High values indicated the perception of high empathic communication. For the present study, the EAP reliability coefficient was equal to 0.630 and the KR-20 was equal to 0.762.

(Perceived) Clarity of information

The fourth set of three items aimed to assess the patient’s perception of the clarity of the information received regarding cancer treatment during the pandemic. (i.e., “*Was the doctor clear in communicating information relating to the management of cancer care during this period?*”). High values indicated the perception of high clarity of information. For the present study, the EAP reliability coefficient was equal to 0.601 and the KR-20 was equal to 0.787.

### 2.4. Procedure

The inclusion/exclusion criteria were applied through both the screening of medical records and a first psychological interview.

Consequently, the ad hoc structured interview was administered by an expert psycho-oncologist (author M.M.) during the initial psychological consultation. Patients’ responses were registered into an electronic report form.

### 2.5. Statistical Analyses

Statistical analyses were performed with the R statistical software [85,86]. with following packages: “lavaan” [87,88], “overlapping” [89], “psych” [90], and “TAM” [91]. Graphical representations were carried out with “graphViz” in “DiagrammeR” package [92].

Considering the novelty of the structured interview, the psychometric properties of its items were assessed. In more detail, given the binary response scale (true/false) as well as the (assumed) unidimensionality of each scale [93,94,95,96], an IRT approach (1PL, Rasch model) was used [93]. Then, the Rasch fit indices were used to assess item psychometric properties: good fit indices suggest that an item fits the Rasch model’s expectations based on item difficulties and subjects’ ability level. In more detail, “infit” and “outfit” were computed given both their sensitivity to unexpected responses and the non-dependence from the sample size. The “infit” detects unexpected responses to items that are close to a person’s trait level. The “outfit” detects greater unexpected responses to items that are far from the subject’s trait level. The recommended values for “infit” and “outfit” is 1 and they sound not be lower than 0.7 or exceed 1.4 [97,98]. Values lower than 0.7 suggest the presence of redundancy among the pool of considered items (namely, overfit). Values above 1.4 indicate the presence of unexplained variance among the set of considered items (namely, underfit) [93,97,98].

Once the psychometric properties of the structured interview were assessed, instead of summing the items, the total score of each scale was computed by extracting their factor scores (FSs), which was used for statistical analyses [99,100,101,102]. Indeed, while the “classical” sum of items assumes that all the items have the same importance, and thus the same weight into the measured construct (namely, tau-equivalence), the FS allows each item to have a unique weight for the measured construct, leading each item to differ in importance [101].

In line with previous studies [6], before testing the hypothesized mediation model, preliminary analyses were performed to exclude the potential effect of external variables. First, the Pearson correlation coefficient (*r*) was computed to evaluate the strength of the relationships between variables [79,103]. A correlation value higher than |0.80| suggests the presence of multicollinearity [79,103] and therefore the violation of the assumptions necessary to carry out the subsequent statistical analyses. Second, considering the result of previous studies [59,63,104], a multiple multivariate regression was performed to test the effect of (A) “civil status”, (B) “work status”, and (C) “education” and (D) “localization of tumor” on variables assessed with the structured interview. In more detail, the external variables were considered as predictors, and the domains of the interview were considered as dependent variables. The strength of the effect of each predictor was interpreted using unstandardized beta (β).

**Figure 1:**
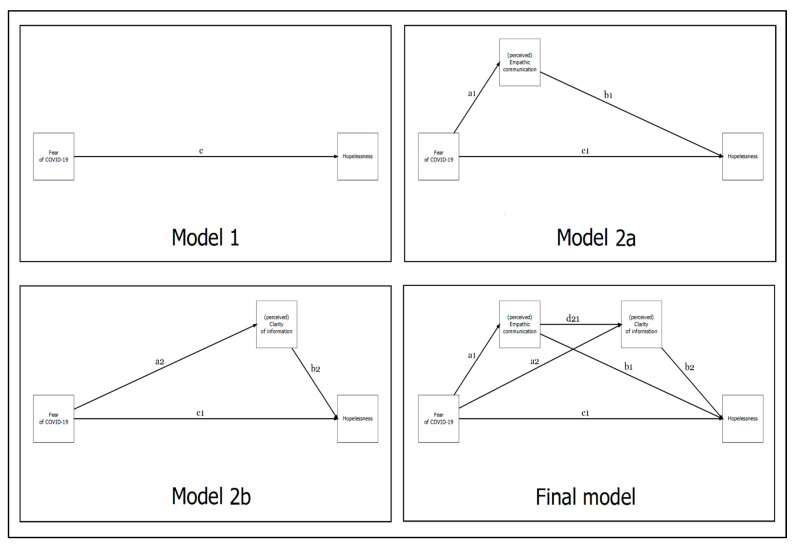
Conceptual graphic representation of each mediation model tested.

Consequently, a path analysis model with observed variables was performed [103,105,106,107,108,109]. In more detail, a sequential mediation model with a single predictor, a single outcome and two mediators were specified [110,111,112]. Considering the continuous nature of FSs, the maximum likelihood (ML) estimator was used to carry out the following statistical analyses. In line with previous studies [6], four steps were followed. *First*, a simple predictor-only model was specified: “fear of COVID-19” (X) was regressed on “hopelessness” (Y) (Figure 1, Model 1). *Second*, a partial mediation model was specified with the effect of a single mediator (i.e., empathic communication): “fear of COVID-19” (X) was regressed on “hopelessness” (Y) through “empathic communication” (M1) (Figure 1, Model 2a). *Third*, an analogous partial mediation model was specified with the effect of the other hypothesized predictor (i.e., clarity of information): “fear of COVID-19” (X) was regressed on “hopelessness” (Y) through “clarity of information” (M2) (Figure 1, Model 2b). *Fourth*, the final sequential multiple mediation model was specified by including all variables: “fear of COVID-19” (X) was regressed on “hopelessness” (Y) through “empathic communication” (M1) and “clarity of information” (M2) (Figure 1, Final Model). All the reported regression coefficients were unstandardized (β).

Finally, considering the small, unbalanced sample between males (*n* = 40) and females (*n* = 50), a multi-group path analysis was not applicable to evaluate gender differences between (A) the two indirect model effect (fear of COVID-19 → empathic communication → clarity of information → hopelessness) and (B) the total model effects. Thus, in line with previous studies [89,113], the effect of the gender (male vs. female) was compared via the overlapping index (η) by overlapping the standard kernel density bootstrap distribution (10,000 replicates) of the standardized model effect parameters [89,114]. The η-index measures the magnitude (effect size) of a phenomenon including similarities and/or differences between groups [89,115,116]. The η-index ranges from 0 (= perfect separation between densities distributions) to 1 (perfect overlap between densities distributions). Thus, it should be interpreted as other normalized effect sizes (i.e., correlation coefficient, *R*^2^, percentage, etc.) [89].

## 3. Results

### 3.1. Psychometric Properties of the Structured Interview

As reported in Table 1, the structured interview provided acceptable fit indices and each item showed acceptable “infit” and “outfit” values. In more detail, considering the “infit” index, despite some items (i.e., item #1 of the “fear of COVID-19” dimension) showed values below the recommended threshold of 0.7 (overfit), none of the 12 items revealed values above the threshold of 1.4 (underfit). Considering the “outfit” index, it could be noted that some items reported values that were under the recommended threshold of 0.7 for overfit; however, none of them revealed values above the threshold of 1.4 for underfit. These results (“infit” and “outfit” values) suggested possible item redundancy. However, the ad hoc structured interview showed acceptable psychometric properties.

### 3.2. Preliminary Analysis

Correlation analyses between FSs showed moderate-to-large associations between variables included in the mediation model. However, none of the reported values exceeded the recommended threshold of |0.80| (Table 2). These results suggested the absence of multicollinearity, thus allowing one to carry out subsequent statistical analyses. In addition, considering continuous external variables, the correlation matrix suggested no statistically significant association between variables included in the path analysis and “age” (Table 2). These results suggested that “age” was not linearly associated with these variables.

The multiple multivariate regression analysis showed no statistically significant effects of predictors (external variables covariates) on the dependent variables. In more detail, controlling for other predictors, no statistically significant effect of the respondents’ (A) *“civil status”* was found on “fear of COVID-19” (β = −0.628, SE = 0.395, *p* = 0.112), “empathic communication” (β = 0.365, SE = 0.316, *p* = 0.248), “clarity of information” (β = −0.184, SE = 0.336, *p* = 0.583), and “hopelessness” (β = 0.041, SE = 0.412, *p* = 0.921).

In addition, controlling for other external variables, no statistically significant effect of the respondents’ (B) “*work status*”, was found on “empathic communication” (β = −0.205, SE = 0.189, *p* = 0.280), “clarity of information” (β = −0.136, SE = 0.165, *p* = 0.407), and “hopelessness” (β = 0.228, SE = 0.213, *p* = 0.283), while a small effect was found for “fear of COVID-19” (β = 0.603, SE = 0.246, *p* = 0.014).

Controlling for other external variables, no statistically significant effect of the respondents’ (C) “e*ducation”* was found on “fear of COVID-19” (β = −0.253, SE = 0.365, *p* = 0.487) and “hopelessness” (β = −0.369, SE = 0.337, *p* = 0.273); while small effects were found for “empathic communication” (β = 0.659, SE = 0.289, *p* = 0.023) and “clarity of information” (β = 0.800, SE = 0.288, *p* = 0.006).

Lastly, controlling for other external variables, no statistically significant effect of the respondents’ (D) “type of cancer” was found on “fear of COVID-19” (β = −0.227, SE = 0.208, *p* = 0.274), “empathic communication” (β = 0.070, SE = 0.156, *p* = 0.652), “clarity of information” (β = 0.109, SE = 0.151, *p* = 0.472), and “hopelessness” (β = −0.219, SE = 0.156, *p* = 0.160).

The results are reported in Table 3.

### 3.3. Sequential Multiple Mediation Model

Partial models

Model 1

Considering the first model (Figure 1, Model 1), the “fear of COVID-19” (X) was positively associated with “hopelessness” (Y), *path c*: β = 0.563 (SE = 0.058), *p* < 0.001. Results are reported in Table 4.

Model 2a

Considering the second model (Figure 1, Model 2a), the “fear of COVID-19” (X) was negatively associated with “empathic communication” (M1), *path a1*: β = −0.524 (SE = 0.061), *p* < 0.001. Moreover, “empathic communication” (M1) negatively predicted “hopelessness” (Y), *path b1*: β = −0.361 (SE = 0.135), *p* = 0.008 suggesting a buffering effect of “empathic communication”.

At the same time, “fear of COVID-19” (X) was directly positively associated with “hopelessness” (Y), *path c1*: β = 0.374 (SE = 0.100), *p* < 0.001.

Furthermore, the total indirect effect (fear of COVID-19 → empathic communication → hopelessness) was statistically significant: β = 0.189 (SE = 0.076), *p* = 0.013. Lastly, the total model effect was statistically significant: β = 0.563 (SE = 0.058), *p* < 0.001, thus suggesting a partially mediated path. The results are reported in Table 4.

Model 2b

Considering the third model (Figure 1, Model 2b), the “fear of COVID-19” (X) was negatively associated with “clarity of information” (M2), *path a2*: β = −0.365 (SE = 0.076), *p* < 0.001. Moreover, “clarity of information” (M2) negatively predicted “hopelessness” (Y), *path b2*: β = −0.350 (SE = 0.093), *p* < 0.001, also suggesting a buffering effect of “clarity of information”.

At the same time, “fear of COVID−19” (X) was directly positively associated with “hopelessness” (Y), *path c1*: β = 0.435 (SE = 0.074), *p* < 0.001.

Furthermore, the total indirect effect (fear of COVID-19 → clarity of information → hopelessness) was statistically significant: β = 0.128 (SE = 0.050), *p* = 0.010. Lastly, the total model effect was statistically significant: β = 0.563 (SE = 0.058), *p* < 0.001, thus suggesting a partially mediated path. The results are reported in Table 4.

Full model

Final model (Model 3)

**Figure 2:**
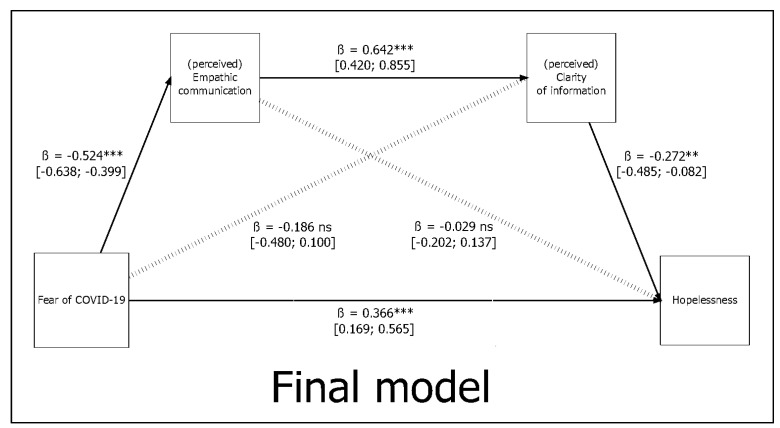
Graphical representation of the final (full) mediation model. ** *p* < 0.010; *** *p* < 0.001; ns = statistically non-significant (*p* > 0.050). Note: continuous lines represent statistically significant relationships and dotted lines represent statistically non-significant relationships. Reported beta coefficients (β) are unstandardized; in square brackets, both lower and upper limits of the confidence interval at 95% are reported.

Considering the fourth model (Figure 1, final model, and Figure 2), “fear of COVID-19” (X) was negatively associated with “empathic communication” (M1), *path a1*: β = −0.524 (SE = 0.061), *p* < 0.001. At the same time, “fear of COVID-19” (X) was not statistically associated with “clarity of information” (M2), *path a2*: β = −0.029 (SE = 0.086), *p* = 0.739 *ns.*

However, “empathic communication” (M1) was positively associated with “clarity of information” (M2), *path d21*: β = 0.642 (SE = 0.110), *p* < 0.001.

Simultaneously, “empathic communication” (M1) was not statistically associated with “hopelessness” (Y), *path b1*: β = −0.186 (SE = 0.148), *p* = 0.207 *ns*. At the same time, “clarity of information” (M2) was negatively associated with “hopelessness” (Y), *path b2*: β = −0.272 (SE = 0.101), *p* = 0.007.

Finally, “fear of COVID−19” (X) was still positively associated with “hopelessness” (Y), *path c1*: β = 0.366 (SE = 0.101), *p* < 0.001.

Furthermore, an examination of the three indirect paths was performed. The *first* total indirect effect (fear of COVID-19 → empathic communication → hopelessness) was *not* statistically significant: β = 0.098 (SE = 0.079), *p* = 0.219 *ns*. In line with these results, the *second* total indirect effect (fear of COVID-19 → clarity of information → hopelessness) was *not* statistically significant: β = 0.008 (SE = 0.027), *p* = 0.771 *ns*. Instead, the *third* total indirect effect (fear of COVID-19 → empathic communication → clarity of information → hopelessness) was statistically significant: β = 0.091 (SE = 0.038), *p* = 0.016.

Lastly, the two total model effects were also examined. The first indirect total model effect (considering each relationship between variables *without* the path from “fear of COVID-19” → “hopelessness”) was statistically significant: β = 0.197 (SE = 0.078), *p* = 0.012. The second total model effect (considering each relationship between variables *plus* the path from “fear of COVID-19” → “hopelessness”) was statistically significant: β = 0.563 (SE = 0.059), *p* < 0.001, thus suggesting a partially mediated path.

The results are reported in Table 5.

### 3.4. Overlapping the Total Model Effects

**Figure 3:**
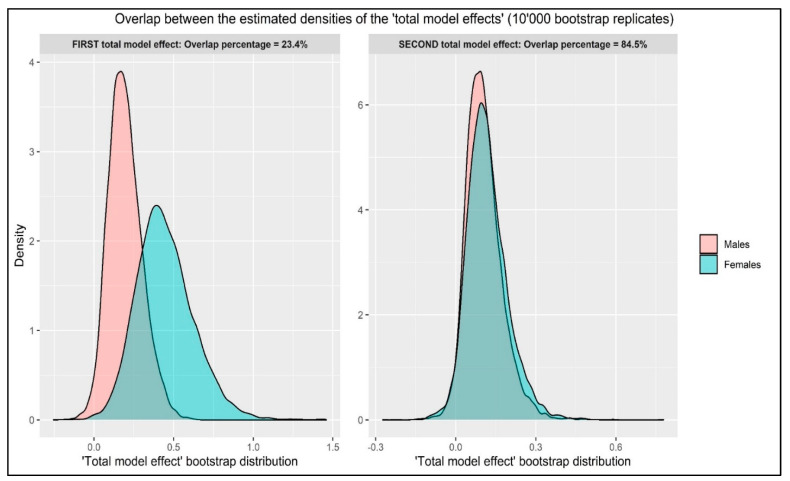
The overlap between the estimated densities of the two “total model effect” between males and females. *Note*: first *indirect* total model effect: each relationship between variables *without* the path from “fear of COVID-19” → “hopelessness”. Second total model effect: each relationship between variables *plus* the path from “fear of COVID-19” → “hopelessness”.

The first *indirect* total model effect (each relationship between variables *without* the path from “fear of COVID-19” → “hopelessness”) was equal to β = 0.164 ((SE = 0.070), *p* = 0.019) for males and it was equal to β = 0.324 ((SE = 0.115), *p* = 0.005) for females. Consequently, the η-index revealed considerable separation between the estimated densities bootstrapped distribution of the “(indirect) total model effect” for both males and females: η = 0.234 (23.4%) with a consequential separation index (“1-η”) of 0.766 (76.6%) (Figure 3).

Considering the second total model effect (each relationship between variables *plus* the path from “fear of COVID-19” → “hopelessness”), it was equal to β = 0.615 ((SE = 0.095), *p* < 0.001) for males and it was equal to β = 0.522 ((SE = 0.082), *p* < 0.001) for females. Consequently, the η-index revealed a substantial overlap between the estimated densities’ bootstrapped distribution of the “total model effect” for both males and females: η = 0.845 (84.5%) with a consequential separation index (“1-η”) of 0.155 (15.5%) (Figure 3).

## 4. Discussion

The COVID-19 virus has shown high mortality rates and symptom severity, putting a strain on the endurance of health care resources around the world [1,2,3,12,117]. As a consequence, the general population has started presenting anxiety and fear related to this illness and its potential mortality [6,118]. Such negative feelings are highly present in clinical populations at greater risk of developing life-threatening COVID-19 symptoms as well, such as oncological patients [14]. Indeed, members of this clinical population, who already present high levels of distress related to their medical condition, have been reporting both fear and an associated feeling of hopelessness regarding the pandemic, their medical condition, the future [9,37,45,119] and their care management [50]. For example, they reported being scared of COVID-19 contagion as well as any of its possible repercussions on cancer treatment (i.e., delays in therapies) [45,47,50]. However, a good doctor–patient communication based on PCC seems to contribute to the reduction in distress and to the improvement of oncological patients’ psychological well-being [58,72,74].

Consequently, the present study aimed at testing if empathy and clarity of communication may buffer the adverse relation between fear of COVID-19 and hopelessness feelings experienced by oncological patients during the COVID-19 pandemic.

A series of path analysis models were consecutively tested. Model 1 showed the existence of a positive relationship between fear of COVID-19 and hopelessness: higher fear of COVID-19 was associated with higher hopelessness experienced by patients.

Model 2a and Model 2b, respectively, tested the mediating (buffering) role of PCC variables, i.e., (perceived) empathic communication and (perceived) clarity of information showing a negative association between them, and both the fear of COVID-19 and hopelessness.

Lastly, Model 3 tested the conjoint sequential effect [103] of empathy and clarity in fully mediating the effect of fear of COVID-19 on hopelessness. These findings suggest that negative feelings due to an external/contextual situation (such as fear of COVID-19) might activate a “need for cognitive closure” [120,121,122] towards the “outside”, which might lead the patient to perceive the doctor’s communication as less empathic and/or clear [64,65,66,67,68] and to experience greater levels of hopelessness [123,124]. Instead, when intense feelings of fear of COVID-19 are not experienced, the “need for cognitive closure” would not be activated, leading the patient to perceive the doctor’s communication as more empathic and/or clear [69,125,126]. In other words, these findings show that a PCC characterized by empathy and clarity would buffer the adverse effects of fear of COVID-19 on hopelessness.

Interestingly, the overlap analysis showed the separation level of the model effects distributions between males and females. Overall, the results showed that the complete model was similar between males and females (overlap 85%). However, the total indirect effect was different between males and females (23% overlap). In particular, the mediated effects were stronger among females, suggesting that the PCC would have a greater impact among females.

These results are in line with the scientific literature [57,59,62,69,70], which show the empathy and clarity of the doctors’ communication to play a central role in medical consultations [62,64,65,66,67,68,127]. Specifically, concerning empathy, patients with cancer explicitly report that they prefer doctors who take their emotions and personal concerns into account while providing sensitive information [62]. Moreover, the oncologists’ use of empathic language would increase patients’ satisfaction with the visit, foster a better relationship with the physician and increase patients’ trust in the physician [64,65,66,67,68,69,70]. Therefore, oncological patients would be more satisfied when their doctors listen and reassure them while taking into account their emotional needs [73].

Moreover, a lack of clear information (i.e., doctors use jargon) would increase the patients’ level of anxiety and contribute to worsening the doctor–patient relationship. Furthermore, unclear information would lead the patients to experience uncertainty, which represents a strong predictor of emotional distress in both patients with cancer and their family members [62,64,65,66,67,68]. The importance of this communicative aspect is highlighted by the fact that patients’ preferences of medical treatments and their decision-making processes are influenced by the information shared by doctors [128,129]. Therefore, patients should receive clear information so that they can have a better understanding of their medical condition and their prognosis [73].

Indeed, in line with findings of previous research, the provision of clear and understandable information would decrease the patients’ level of anxiety, and increase their sleep quality, appetite, hope for the future, and satisfaction with the visit and with the physician [57,64,65,66,67,68].

Conversely, a lack of communication skills might even worsen rather than improve the psychological well-being and adherence to treatment recommendations of patients suffering from cancer and other chronic conditions [130]. For example, some types of reassurance, i.e., spontaneous reassurance or reassurance provided before the patients share their concerns might increase anxiety symptoms, worries, and feelings of uncertainty [131].

Therefore, good communication skills among doctors are crucial in building a trustworthy doctor–patient relationship that not only helps in therapeutic success but also increases their job satisfaction. Unfortunately, not many doctors are naturally blessed to have good communication skills and there is a necessity for formal training [57,132,133].

These results seem to further prove how empathy and clarity of communication in doctor–patient dialogues may buffer patients’ development of psychological symptoms [62,134]. The innovation and usefulness of these results consist of the fact that it seems to be the first time that the buffering effect of the two aforementioned communicative elements is proved in relation to COVID-19-related negative feelings. Consequently, physicians could take into account these communicative functions when interacting with patients since they seem to “shield” their psychological well-being [71,72,73]. Furthermore, it is important to highlight that on the basis of this and previous results [57,132,133,135], it may be possible to structure communication skills training for health personnel working with oncological patients. Future studies should be conducted in this regard.

Some limitations of this research should be listed. First, a relatively small sample was used. However, some studies showed that even 60 cases could provide an accurate estimation of path analysis models [79,136], suggesting that the enrolled sample could be considered enough. Second, the social desirability of respondents may have influenced their answers to the structured clinical interview. Additionally, despite the fact that administered structured interview showed good psychometric properties with good “infit” and “outfit” indices, the unfavorable environmental conditions (i.e., hygienic reasons) did not allow the use of any further assessment tool such as self-report questionnaires [8]. Still, the clinical interview allowed one to simultaneously investigate the three basic components for effective communication: verbal (i.e., the content of the message), non-verbal; (i.e., body language such as posture, gesture, facial expression, and spatial distance), and para-verbal (i.e., including tone, pitch, pacing and volume of the voice). Indeed, the verbal component constitutes only ten percent of the message delivered. Non-verbal and para-verbal components would, instead, contribute to ninety percent of the total message delivered, influencing, and mirroring important treatment outcomes patient’s satisfaction, adherence [137]. Third, despite the advantage of being a short measure, the ad hoc structured interview employed in this study only included three questions for each aspect of the doctor–patient communication. Future studies should, therefore, make use of measures comprising more items to allow for a better understanding of the phenomenon. Fourth, this study aimed to focus exclusively on empathy and clarity as elements of doctor–patient communication, and future research should focus and test other features of PCC (i.e., listening, reframing) to increase the physicians’ ability to manage difficult clinical encounters, so to improve treatment outcomes. Fifth, due to the pandemic emergency, it was not possible to enroll the sample by stratifying it based on the disease/treatment stage. These variables should be, therefore, carefully taken into account in future studies. Lastly, due to the correlational/observational nature of the research design and in line with the purpose of the study, it was possible to test the predictive relationships among variables but not to establish a causal relationship among them [138].

Still, to our knowledge, this study represents the first that highlights how PCC based on doctors’ empathic and clear communication might have a buffering effect on the relationship between fear of COVID-19 and lack of hope experienced by oncological patients during the pandemic.

Moreover, a methodological peculiarity is the use of factor scores that allowed each item to have a realistic weight for the measured construct.

Future research should further test the buffering effect of empathy and clarity of information in the relationship between fear and hopelessness oncological patients in different settings and cultures. Additionally, research that aims to evaluate the short and long-term benefits of effective doctor–patient communication on diverse life domains and the overall perceived quality of life of patients with cancer is warranted.

Furthermore, other studies should investigate the possible differences between PCC conducted face-to-face compared with that provided via digital tools. In addition, other variables should be included in future research, such as the patients’ levels of distress and/or the presence of depressive symptoms, to further understand the mechanism underlying the benefits of empathy and clarity (alone or together with other important elements of doctor–patient communication) on this population of patients.

## 5. Conclusions

The present research supports the efficacy of a PCC in the oncological setting, with a specific focus on the role played by empathy and clarity of information in increasing patients’ well-being. Specifically, this study showed the buffering effect of the doctors’ communication on the relationship between the patients’ fear of COVID-19 and hopelessness for the future. Overall, the findings of this study contribute to a better understanding of the importance of physicians’ empathic communication with oncological patients, providing evidence of its mediating role in the context of the COVID-19 pandemic.

## Figures and Tables

**Table 1:** Infit, outfit, and item difficulty of each item of the structured interview.

	Infit	Outfit	Xsi (SE)
Fear of COVID-19			
Considering your oncological disease, are you afraid of COVID-19?	0.726	0.501	1.180 (0.328)
Considering your oncological disease, are you anxious about COVID-19?	0.788	0.620	0.138 (0.319)
Considering your oncological disease, are you preoccupied with COVID-19?	0.851	0.680	0.966 (0.326)
Empathic communication			
Was the doctor empathetic in communicating the management of cancer care during this period?	0.998	0.983	−0.577 (0.288)
Was the doctor reassuring in communicating the management of cancer care during this period?	0.876	0.667	−1.439 (0.302)
Was the doctor warm in communicating the management of cancer care during this period?	0.984	0.823	−1.439 (0.302)
Clarity of information			
Was the doctor precise in communicating information relating to the management of cancer care during this period?	0.792	0.612	−1.544 (0.311)
Was the doctor explicit in communicating information relating to the management of cancer care during this period?	0.911	0.783	−1.742 (0.317)
Was the doctor clear in communicating information relating to the management of cancer care during this period?	1.072	1.241	−2.162 (0.332)
Hopelessness			
Considering your oncological disease, did the future seem hopeless to you?	0.953	0.848	2.024 (0.329)
Considering your oncological disease, did the future seem difficult to face?	0.863	0.772	1.312 (0.310)
Considering your oncological disease, did the future seem more negative than positive to you?	0.935	0.768	1.917 (0.326)

**Table 2:** Bivariate correlation analysis between variables involved in the path analysis and age.

	Fear of COVID-19	Empathic Communication	Clarity of Information	Hopelessness	Age
Fear of COVID-19	-				
Empathic communication	−0.691 ***	-			
Clarity of information	−0.475 ***	0.658 ***	-		
Hopelessness	0.689 ***	−0.651 ***	−0.583 ***	-	
Age	0.176 ^§^	−0.177 ^§^	−0.122 ^§^	0.169 ^§^	-

Note: *** *p* < 0.001; ^§^ *p* > 0.050 *ns.*

**Table 3:** Preliminary analysis: multiple multivariate regression analysis among external variables and dependent variables: summary of the parameter estimates (beta) with 95% confidence intervals.

External Variable	Dependent Variable	Β *	β (SE)	95%CI (L U)	*z*-Value	*p*-Value
Civil status	Fear of COVID-19	−0.164	−0.628 (0.395)	(−1.403; 0.146)	−1.590	*p* = 0.112
	Empathic communication	0.126	0.365 (0.316)	(−0.254; 0.985)	1.155	*p* = 0.248
	Clarity of information	−0.063	−0.184 (0.336)	(−0.842; 0.474)	−0.549	*p* = 0.583
	Hopelessness	0.013	0.041 (0.412)	(−0.767; 0.848)	0.099	*p* = 0.921
Work status	Fear of COVID−19	0.242	0.603 (0.246)	(0.121; 1.085)	2.450	*p* = 0.014
	Empathic communication	−0.108	−0.205 (0.189)	(−0.576; 0.167)	−1.080	*p* = 0.280
	Clarity of information	−0.071	−0.136 (0.165)	(−0.459; 0.186)	−0.829	*p* = 0.407
	Hopelessness	0.112	0.228 (0.213)	(−0.189; 0.645)	1.073	*p* = 0.283
Education	Fear of COVID−19	−0.068	−0.253 (0.365)	(−0.968; 0.461)	−0.695	*p* = 0.487
	Empathic communication	0.233	0.659 (0.289)	(0.092; 1.226)	2.278	*p* = 0.023
	Clarity of information	0.279	0.800 (0.288)	(0.234; 1.365)	2.772	*p* = 0.006
	Hopelessness	−0.121	−0.369 (0.337)	(−1.028; 0.291)	−1.096	*p* = 0.273
Type of cancer	Fear of COVID−19	−0.116	−0.227 (0.208)	(−0.634; 0.180)	−1.093	*p* = 0.274
	Empathic communication	0.047	0.070 (0.156)	(−0.235; 0.375)	0.450	*p* = 0.652
	Clarity of information	0.072	0.109 (0.151)	(−0.187; 0.404)	0.719	*p* = 0.472
	Hopelessness	−0.137	−0.219 (0.156)	(−0.524; 0.086)	−1.405	*p* = 0.160

Note: β * = standardized beta; β = unstandardized beta; 95%CI = 95% confidence intervals for the unstandardized beta.

**Table 4:** Summary of parameter estimates (beta) with 95% confidence intervals for key pathways tested for each model: Model 1; Model 2a; Model 2b (Figure 1).

Path		β *	β (SE)	95%CI (L U)	*z*-Value	*p*-Value	*R* ^2^
Model 1							
Fear of COVID-19 (X) → Hopelessness (Y)	(*c*)	0.689	0.563 (0.058)	(0.448; 0.676)	9.625	*p* < 0.001	0.475
Model 2a							
Fear of COVID-19 (X) → Empathic communication (M1)	(*a1*)	−0.691	−0.524 (0.061)	−0.639 −0.401	−8.585	*p* < 0.001	0.478
Empathic communication (M1) → Hopelessness (Y)	(*b1*)	−0.335	−0.361 (0.135)	−0.621 −0.089	−2.666	*p* = 0.008	0.534
Fear of COVID-19 (X) → Hopelessness (Y)	(*c1*)	0.458	0.374 (0.100)	0.183 0.576	3.723	*p* < 0.001	
Indirect effect of X on Y via M1	(a1*b1)	0.231	0.189 (0.076)	0.046 0.350	2.478	*p* = 0.013	
Total effect X on Y		0.689	0.563 (0.058)	0.447 0.677	9.640	*p* < 0.001	
Model 2b							
Fear of COVID-19 (X) → Clarity of information (M2)	(*a2*)	−0.475	−0.365 (0.076)	−0.512 −0.214	−4.802	*p* < 0.001	0.225
Clarity of information (M2) → Hopelessness (Y)	(*b2*)	−0.330	−0.350 (0.093)	−0.543 −0.177	−3.756	*p* < 0.001	0.559
Fear of COVID-19 (X) → Hopelessness (Y)	(*c1*)	0.533	0.435 (0.074)	0.281 0.571	5.883	*p* < 0.001	
Indirect effect of X on Y via M2	(a2*b2)	0.156	0.128 (0.050)	0.050 0.244	2.566	*p* = 0.010	
Total effect X on Y		0.689	0.563 (0.058)	0.445 0.675	9.673	*p* < 0.001	

Note: β* = standardized beta; β = unstandardized beta; 95%CI = 95% confidence intervals for the unstandardized beta; *R*^2^ = explained variance.

**Table 5:** Summary of parameter estimates (beta) with 95% confidence intervals for key pathways tested for the final model: model 3 (Figure 2).

Path		β *	β (SE)	95%CI (L U)	*z*-Value	*p*-Value	*R* ^2^
Fear of COVID-19 (X) → Empathic communication (M1)	(*a1*)	−0.691	−0.524 (0.061)	(−0.638; −0.399)	−8.584	*p* < 0.001	0.478
Fear of COVID-19 (X) → Clarity of information (M2)	(*a2*)	−0.037	−0.029 (0.086)	(−0.202; 0.137)	−0.333	*p* = 0.739	0.438
Empathic communication (M1) → Clarity of information (M2)	(*d21*)	0.632	0.642 (0.110)	(0.420; 0.855)	5.830	*p* < 0.001	
Empathic communication (M1) → Hopelessness (Y)	(*b1*)	−0.173	−0.186 (0.148)	(−0.480; 0.100)	−1.262	*p* = 0.207	
Clarity of information (M2) → Hopelessness (Y)	(*b2*)	−0.256	−0.272 (0.101)	(−0.485; −0.082)	−2.692	*p* = 0.007	
Fear of COVID−19 (X) → Hopelessness (Y)	(*c1*)	0.448	0.366 (0.101)	(0.169; 0.565)	3.626	*p* < 0.001	0.571
Indirect effect of X on Y via M1	(a1*b1)	0.119	0.098 (0.079)	(−0.055; 0.263)	1.228	*p* = 0.219	
Indirect effect of X on Y via M2	(a2*b2)	−0.010	0.008 (0.027)	(−0.033; 0.075)	0.291	*p* = 0.771	
Indirect effect of X on Y via M1 and M2	(a1*d21*b2)	0.112	0.091 (0.038)	(0.027; 0.176)	2.410	*p* = 0.016	
Total indirect effect		0.241	0.197 (0.078)	(0.056; 0.362)	2.523	*p* = 0.012	
Total effect X on Y		0.689	0.563 (0.059)	(0.447; 0.678)	9.564	*p* < 0.001	

Note: β * = standardized beta; β = unstandardized beta; 95%CI = 95% confidence intervals for the unstandardized beta; R^2^ = explained variance.

## Data Availability

The data presented in this study are available on request from the corresponding author. The data are not publicly available due to privacy restrictions.
